# Chloroplast Genome Sequence of *Artemisia scoparia*: Comparative Analyses and Screening of Mutational Hotspots

**DOI:** 10.3390/plants8110476

**Published:** 2019-11-06

**Authors:** Shabina Iram, Muhammad Qasim Hayat, Muhammad Tahir, Alvina Gul, Abdullah, Ibrar Ahmed

**Affiliations:** 1 Department of Plant Biotechnology, Atta-Ur-Rahman School of Applied Biosciences (ASAB), National University of Sciences and Technology (NUST), H-12 Islamabad 44000, Pakistan; muhammad.tahir@asab.nust.edu.pk (M.T.); alvina_gul@asab.nust.edu.pk (A.G.); 2 Department of Biochemistry, Quaid-i-Azam University, Islamabad 45320, Pakistan; abd.ullah@bs.qau.edu.pk; 3 Alpha Genomics Private Limited, Islamabad 45710, Pakistan; alphagenomics.co@gmail.com

**Keywords:** *Artemisia scoparia*, Asteraceae, chloroplast genome, substitutions, divergence region, taxonomic discrepancies, phylogenetic

## Abstract

*Artemisia* L. is among the most diverse and medicinally important genera of the plant family Asteraceae. Discrepancies arise in the taxonomic classification of *Artemisia* due to the occurrence of multiple polyploidy events in separate lineages and its complex morphology. The discrepancies could be resolved by increasing the genomic resources. *A. scoparia* is one of the most medicinally important species in *Artemisia*. In this paper, we report the complete chloroplast genome sequence of *Artemisia scoparia*. The genome was 151,060 bp (base pairs), comprising a large single copy (82,834 bp) and small single copy (18,282 bp), separated by a pair of long inverted repeats (IRa and IRb: 24,972 bp each). We identified 114 unique genes, including four ribosomal RNAs, 30 transfer RNAs, and 80 protein-coding genes. We analysed the chloroplast genome features, including oligonucleotide repeats, microsatellites, amino acid frequencies, RNA editing sites, and codon usage. Transversion substitutions were twice as frequent as transition substitutions. Mutational hotspot loci included *ccsA-ndhD*, *trnH-psbA*, *ndhG-ndhI*, *rps18-rpl20*, and *rps15-ycf1*. These loci can be used to develop cost-effective and robust molecular markers for resolving the taxonomic discrepancies. The reconstructed phylogenetic tree supported previous findings of *Artemisia* as a monophyletic genus, sister to the genus *Chrysanthemum*, whereby *A. scoparia* appeared as sister to *A. capillaris*.

## 1. Introduction

The genus *Artemisia* L. includes over 500 species and occupies the top position in the family *Asteraceae* in terms of its bio-prospection [1]. Members of this genus are mainly hairy shrubs and herbs with a cosmopolitan distribution in arid and semi-arid habitats [2]. Western and central Asia is considered its centre of origin in the northern hemisphere, although some species are also found in the southern hemisphere [3]. Several species of *Artemisia* have wide and varied medicinal applications in the pharmaceutical industry, as well as in folk remedies [1].

The vernacular name of *Artemisia scoparia* Waldst. et. Kit (red stem wormwood) is Jhahoo or Jaukay in Pakistan [2]. It is a branched perennial herb with a bitter aroma [2]. The herb has a wild distribution in Southwest Asia and central Europe [4]. In northern Pakistan, *A. scoparia* is found growing up to an altitude of 4000 m in the summer season along the field boundaries, on stony grounds, rural tracks, and sandy soils of barren areas after rainfall [5].

Recently, *A. scoparia* has received immense attention due to its potential therapeutic impacts on indigenous communities. This species has been reported to contain anti-viral [6], anti-cancer [7], anti-inflammatory [8], anti-allergic [9], anti-oxidant, anti-malarial, insecticidal [10], anti-microbial [11], anti-hypertensive [12], and anti-obesity [13] properties. This species also has renal-protective [5], hepato-protective [14], hypo-lipidemic [15], and urease inhibitory [16] properties. Moreover, it has been utilized for curing Alzheimer’s disease [17].

The chloroplast (cp) genome in most of the plants has a characteristic quadripartite and circular structure, in which a small single copy region (SSC) and a large single copy region (LSC) are separated by a pair of large inverted repeats (IRs) [18,19]. Linear chloroplast genomes are also reported in some species [20]. The size of the cp genome ranges from 107 to 218 kb [21]. The cp genome is conservative in nature and is mostly inherited maternally [21]. Chloroplast genome polymorphism has been used in phylogenetic reconstructions from population genetics [22] to investigate deep divergence at a genera and family level [23,24,25,26]. The slow evolving nature and maternal inheritance of the chloroplast genome in comparison to the nuclear genome make it suitable for such studies [21]. Therefore, the chloroplast genome structure can be exploited for the taxonomic classification, inferring of phylogeny, and molecular barcoding of the medicinal plants like *A. scoparia*. Knowledge about the chloroplast genome structure is also helpful in the transformation of foreign genes to obtain high protein expression [21,27,28].

Based on its extensive medicinal applications and ecological value, it is important to further explore the genetic and phylogenetic characteristics of *A. scoparia*. Highly efficient molecular markers are required to select pharmaceutically efficient germplasm for large-scale breeding purposes in *Artemisia* [27]. The acquisition of genomic information can help to develop medicinally potent genetic breeds of the plant. Traditionally, the taxonomic identification of *Artemisia* species has relied on morphological characteristics. However, controversies with regards to the taxonomic relationships have arisen due to the plastic nature, complex genetics, and polyploidy of *Artemisia* species, limiting the role of morphological characteristics as the sole factor for classification [29,30]. The development of high-resolution genetic markers is important for fostering attempts regarding species identification, inferring phylogeny, and improving the medicinal value of *A. scoparia*.

In this study, we de novo assembled the chloroplast genome of *A. scoparia* and compared the chloroplast genome features of nine *Artemisia* species. We analysed the codon usage, amino acid frequency, RNA editing sites, and repeats in the *A. scoparia* genome. We also identified twenty polymorphic loci which might be appropriate for the design of authentic, cost-effective, and high-efficacy molecular markers. The phylogeny was inferred to understand the phylogenetic position of *A. scoparia*. The findings of our study are expected to be useful in phylogenetics, population genetics, and genetic engineering studies of *A. scoparia*.

## 2. Results

### 2.1. Chloroplast Genome Features of A. scoparia

Hiseq2500 generated 6.6 GB sequencing data that contained 18.54 million reads from the paired-end run. This whole genome sequence data was used to de novo assemble the chloroplast genome of *A. scoparia* with an average coverage depth of 64 ×. The *Artemisia scoparia* chloroplast genome had a quadripartite structure with a length of 151,060 bp (base pairs). The chloroplast genome contained an LSC (82,834 bp) region and an SSC (18,282 bp) region, which were separated by two copies of IRs (IRa and IRb: 24,972 bp each).

**Figure 1:**
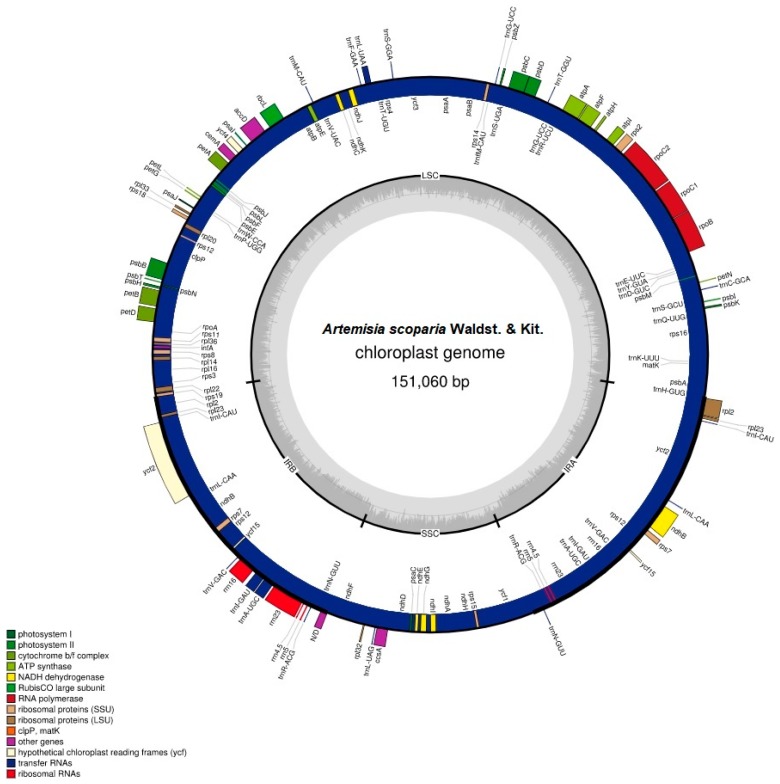
Circular map of the *Artemisia scoparia* chloroplast genome. The genes present outside of the circle are transcribed anti-clockwise, while those inside the circle are transcribed clockwise. Large single copy (LSC), inverted repeat (IRa, IRb), and small single copy (SSC) regions are indicated. The dashed grey color of the inner circle shows the GC content, whereas the lighter gray color shows the AT content. Color of genes was assigned based on their functions.

The chloroplast genome contained 114 unique genes in which 18 genes were also duplicated in IRs, except for the pseudogene copy of *ycf1^Ψ^*. Among 114 unique genes, 30 were tRNA genes, 4 were rRNA genes, and 80 were protein-coding genes. Eighteen genes that were duplicated in IRs comprised 4 rRNA genes, 7 tRNA genes, and 7 protein-coding genes. We also found 18 intron-containing genes that included 6 tRNA genes and 12 protein-coding genes. Among the 18 genes, 16 genes contained one intron, whereas 2 genes (*ycf3* and *clpP*) contained two introns. The *rps12* gene was a trans-splicing gene in which the 5′ end existed in the LSC as a single copy, whereas the 3′ existed in the IRs regions in duplicate. The *ycf1* gene started from the IRa region and extended into the SSC region, and thus left a truncated copy of *ycf1^Ψ^* at the border of the IRb region. The arrangement of genes in the chloroplast genome has been shown in Figure 1. The gene content of the genome has been shown, along with their function, in Table S1.

The chloroplast genome of *A. scoparia* is AT-rich. The complete chloroplast genome is comprised of 62.5% AT content and 37.5% GC content. The GC content of the three regions varies in the genome. The IR regions contain a high GC content (43.1%), compared to the LSC (35.6%) and SSC (30.8%) regions. The high GC content of IRs is due to the presence of high GC-comprising genes: rRNAs (55%) and tRNAs (52.7%). The genomic features have been provided in detail in Table 1.

### 2.2. Codon Usage and Amino Acid Frequency

Codon usage analyses revealed a high encoding of codons that contained A or T at the 3′ end among synonymous codons compared to codons that ended with C or G (Table S2). The amino acid frequency analyses revealed an abundance of leucine and isoleucine, whereas cysteine was the least abundant amino acid (Figure S1).

### 2.3. RNA Editing Sites

The predictive RNA editor for plant chloroplast genes (PREP-cp) predicted 51 post-transcriptional RNA editing modifications in 21 protein-coding genes. Most of the RNA editing sites were found in *ndhB* (9 editing sites) and *ndhD* (6 editing sites), whereas *matK*, *rpoC1*, and *accD* contained four RNA editing sites each. All the changes took place on the first and second nucleotide of codons. However, the conversion rate at the first nucleotide was observed to be about four times higher than that at the second nucleotide. Most of the RNA editing sites resulted in the conversion of serine to leucine and phenylalanine. Moreover, most of the conversion led to hydrophobic amino acids, including methionine, phenylalanine, valine, leucine etc. (Table S3).

### 2.4. Comparison of Genus Artemisia Genomes and the Contraction and Expansion of Large Inverted Repeats

We compared the chloroplast genome features of nine *Artemisia* species and also evaluated the phenomenon of the IR contraction and expansion. The size of the complete chloroplast genome varied from 151,011 bp (in *A. fukudo*) to 151,318 bp (in *A. gmelinii*); the LSC region varied from 82,740 bp (in *A. frigida*) to 83,061 bp (in *A. gmelinii*); the SSC region varied from 18,282 bp (in *A. scoparia*) to 18,423 bp (in *A. absinthium*); and each IR varied from 24,892 bp (in *A. absinthium*) to 24,972 bp (in *A. frigida* and *A. scoparia*) (Table S4).

**Figure 2:**
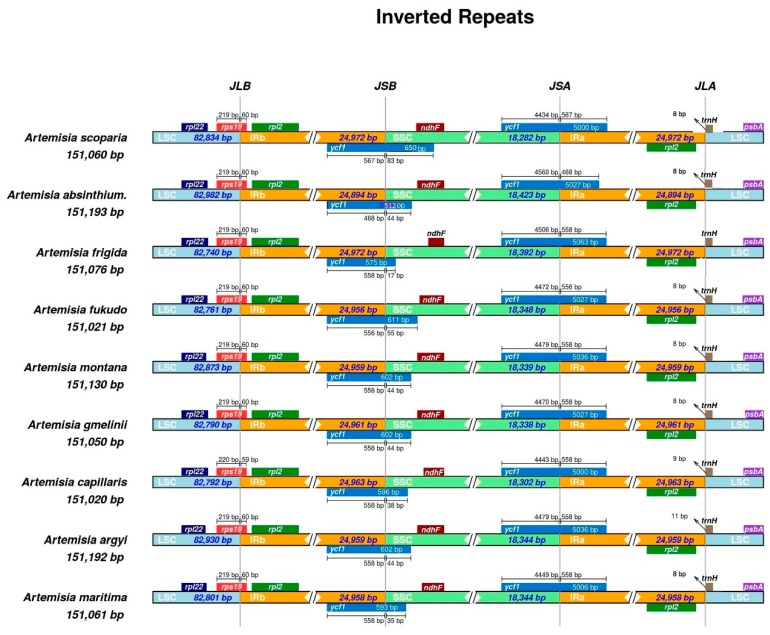
Details of the contraction and expansion of inverted repeats at junction sites. For all plant species, positive strand genes are represented at the top, from right to left, on the corresponding track, whereas negative strand genes are illustrated on the lower side of the track, from left to right. Arrows depict the distance between the start and end of a gene from the junction site. Scale bar present above or below the genes extending from one region to another illustrates the number of base pairs to which genes join in that region. JSA (SSC/IRa), JSB (IRb/SSC), JLA (IRa/LSC), and JLB (IRb/LSC) indicate the junction sites between two corresponding regions of a genome. The plotted genes and distances surrounding the junction site are a scaled projection of the genome.

The IR contraction and expansion were also evaluated among nine species of the genus *Artemisia*. The pseudogene of *rps19^Ψ^* was observed at the junction of LSC and Ira, whereas the pseudogene of *ycf1^Ψ^* was observed at the junction of IRb and SSC. The *rps19* gene integrated from the LSC region to the IRb region with 59 to 60 bp extending into the IRb region, leaving *rps19^Ψ^* at junctions of IRa and LSC. The size of *ycf1^Ψ^* varied from 512 bp (in *A. absinthium*) to 650 bp (in *A. scoparia*) and integrated from the IRb region to SSC region with 17 bp (in *A. frigida*) to 83 bp (in *A. scoparia*). The *ndhF* gene was present at the junction of SSC and IRb completely in the SSC region, but also overlapped with *ycf1^Ψ^* up to 30 bp in *Artemisia scoparia*. The gene *trnH* at the junction of IRa and LSC remained completely in the LSC region, whereas the *rpl2* gene remained completely in the repeated region at the junction of LSC and IRb/IRa. The graphical representation of IR contraction and expansion has been shown in Figure 2.

### 2.5. Microsatellite and Oligonucleotide Repeats Analyses

We used the Perl script of MIcroSAtellite identification tools (MISA) for the identification of microsatellite repeats and detected 58 microsatellite repeats and eight compound repeats. All compound repeats were found in LSC regions. Among the 58 microsatellite repeats, 45 repeats existed in the LSC region, 4 in IR regions, and 9 in the SSC region. The five types of repeats were detected and varied in number, such as Mononucleotide (40) > tetranucleotide (11) > dinucleotide (9) > trinucleotide (5) > pentanucleotide (1). The mononucleotide repeats were made of 10–20 repeat units, dinucleotide repeats were made of 5–7 repeat units, trinucleotide repeats were made of 4–5 repeat units, tetranucleotide repeats were made of 3–4 repeat units, and the pentanucleotide repeat was made of 3 repeat units. All types of repeats were AT-rich (Table 2). The position, type, and region of each microsatellite has been given Table S5.

We used the REPuter program to detect oligonucleotide repeats. The REPuter program detected 14 oligonucleotide repeats, including 10 forward repeats, three palindromic repeats, and one reverse repeat. The size of repeats varied from 30 to 60 bp. The LSC region contained five repeats, IR region contained three repeats, and SSC region contained one repeat. Moreover, three repeats were shared between LSC and IR, one repeat in LSC and SSC, and one repeat in SSC and IR. As per further categorization based on the functional regions of the chloroplast genome, six repeats were observed in intergenic spacer regions, four in coding regions, and two in intronic regions, and two repeat pairs were shared in intronic and intergenic spacer regions (Table 3).

### 2.6. Types of Substitutions and InDels Events

We analysed the types of substitutions and InDel events by using *A. scoparia* as a reference in pairwise alignment with the *A. capillaris* cp genome. We found 88 substitutions in the complete chloroplast genome: 59 substitutions were found in LSC, 23 in SSC, and 6 in IR regions. Among a total of 54 InDels, 42 were in LSC, 9 in SSC, and 3 in IR regions (Table 4).

There were 29 transition substitutions and 59 transversion substitutions. Therefore, we observed a transitions to transversions (Ts/Tv) ratio of 0.49. The Ts/Tv values were 0.49 in the LSC region, 0.55 in the SSC region, and 5 × (five among six were transitions) in IR regions.

### 2.7. Screening of Divergence Regions

**Figure 3:**
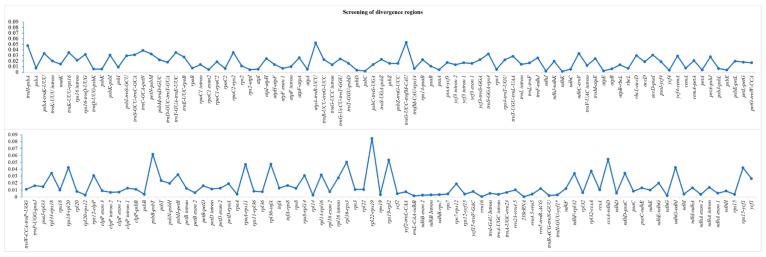
The x-axis shows the regions of the chloroplast genome and the y-axis shows the nucleotide diversity of each region. The regions with 0 nucleotide diversity are not included in the list.

We conducted a multiple sequence alignment of complete chloroplast genomes of nine *Artemisia* species and screened intergenic spacer regions, intronic regions, and protein-coding sequences for the identification of highly diverse regions. The average divergence (π) in complete chloroplast genomes was 0.014. The highest average divergence was recorded for intergenic spacer regions (0.022), followed by introns (0.012) and then protein-coding sequences (0.005). The nucleotide diversity of each region has been shown in Figure 3. We also identified twenty mutational hotspots from intergenic spacer regions that might be adopted as appropriate loci for population genetic and phylogeographic studies. Notable among these loci are *ccsA-ndhD*, *trnH-psbA*, *ndhG-ndhI*, *rps18-rpl20*, and *rps15-ycf1* (Table 5). Among protein-coding genes, *ycf1*, *psbD*, and *accD* exhibited remarkable polymorphism.

### 2.8. Inferring of Phylogeny in the Genus Artemisia and Family Asteraceae

**Figure 4:**
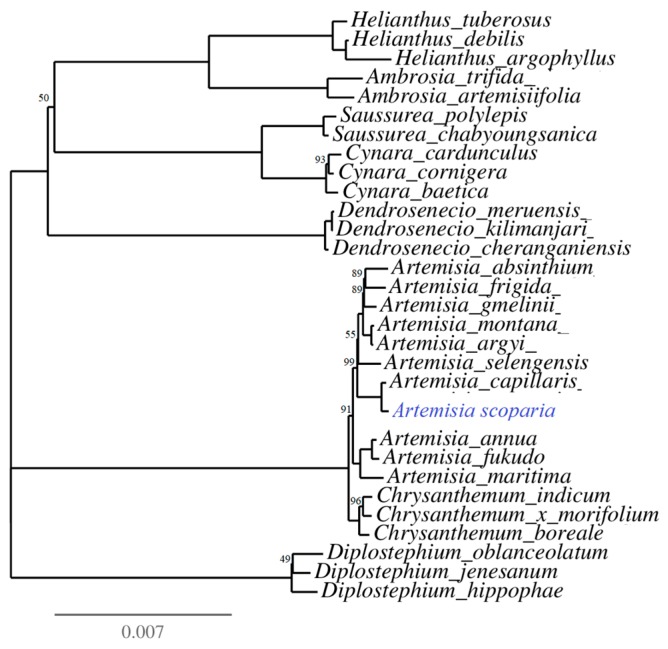
Phylogenetic relationships among 30 species of the family Asteraceae. The number on each node represents the bootstrapping value. The bootstrapping values equal to 100 were not represented on the nodes and omitted from the tree for quality visualization.

The phylogenetic tree was reconstructed for 30 species of the family Asteraceae (Table S6). The phylogeny was inferred based on coding sequences of 77 protein-coding genes using the IQ-tree online program with the best fit model TVM + F + I + G4. The multiple sequence alignment consisted of 57,226 nucleotide sites, in which 53,270 (93.09%) nucleotide sites were constant (invariant sites), 3360 were parsimony informative sites, and 970 showed a distinct pattern. The phylogeny analyses showed *Artemisia* as a monophyletic genus which was sister to the genus *Chrysanthemum*. Moreover, *A. scoparia* was found as sister taxa to *A. capillaris* (Figure 4).

## 3. Discussion

In the current study, we de novo assembled the chloroplast genome of *A. scoparia* and compared its genome with the chloroplast genome of eight publicly available *Artemisia* species. We evaluated the IR contractions and expansion phenomenon in these species, which revealed the origination of the pseudogene *ycf1^Ψ^*. We also analysed types of substitutions, InDels events, and repeats within the genomes.

All nine species of the genus *Artemisia* showed similarity in terms of the gene content, intron content, and composition of GC content. The chloroplast genome of angiosperms is highly conserved and several previous studies have also reported high similarity in chloroplast genome sequences of the same lineage [26,31,32,33,34,35]. However, some studies have reported variation in gene numbers and gain or loss of introns at a genus level, as well as at a family level [36,37,38]. We found a functional *infA* gene, encoding the translation initiation factor, in all species of *Artemisia*, whereas in some other plant lineages, the complete loss or non-function copy of the *infA* gene has been reported [37,38,39].

RNA editing sites are commonly reported in protein-coding genes of the chloroplast. We identified 51 RNA editing sites in 21 protein-coding genes of *A. scoparia*. We observed most of the conversion for serine to leucine and conversion of hydrophilic amino acids into hydrophobic amino acids. Our study is in line with previous studies of other angiosperms in which a similar effect of RNA editing events has been reported [26,36,37].

The contraction and expansion of IR regions, along with the variation in the length of the intergenic spacer region, caused variation in the length of the chloroplast genome, even within same lineages at a family level, as well as at a genus level [26,36,40,41]. The contraction and expansion of the IR regions lead to the duplication of genes, to the loss of one copy of genes, or to the origination of pseudogenes in the chloroplast genome of angiosperms [37,38,42,43]. Our results are also in agreement with these studies and we observed the origination of *ycf1^Ψ^* and *rps19^Ψ^* at the junction of IRb/SSC and LSC/IRa, respectively. The IR contraction and expansion might be helpful in the study of evolutionary patterns [38] and the closely related species show a high resemblance at the junction of the chloroplast genome compared to more diverse species [44]. Our study also agreed with this study and the species of *Artemisia* revealed a closed resemblance at junctions of the chloroplast genome, but this view needs further confirmation in more diverse species (a family level comparison).

We analysed microsatellites and oligonucleotide repeats in the genome of *A. scoparia*. Microsatellites are good markers in studies of population genetics of plant species. Therefore, the identified loci might be suitable for the population genetic studies of *A. scoparia*. The identified microsatellite loci showed an abundance of A/T content, which might be due to the AT-rich composition of the chloroplast genome [36]. In the current study, the mononucleotide repeats were followed by tetranucleotide repeats. However, in other *Artemisia* species, the abundance of dinucleotide repeats was also reported after mononucleotide repeats [45]. In other plant lineages of angiosperm, an abundance of trinucleotide repeats was also found after mononucleotide repeats [36,37]. We found most of the repeats in the LSC region compared to SSC and IR regions. These results are also in agreement with several other studies of angiosperms [36,46,47,48,49].

We also analysed oligonucleotide repeats in the chloroplast genome of *A. scoparia*. Oligonucleotide repeats are considered to produce substitutions, deletion, inversion, addition, and rearrangements within the genome [22,37,39,50,51]. Moreover, these repeats could also be used for the identification of polymorphic loci, as suggested previously [39,51]. Most of the repeats were found in intergenic spacer regions compared to intronic regions and protein-coding sequences. Our results agreed with previous studies of the genus *Artemisia* and other species of angiosperm [26,27,36,40,45,52]. However, protein-coding sequences are also reported to contain the highest number of oligonucleotide repeats [38].

Previous studies reported a higher existence of InDels and substitutions in LSC and SSC regions compared to IR regions [36,39,45,53]. In the current study, an abundance of substitutions and InDels was also observed in LSC and SSC regions compared to IR regions. The most common types of substitutions that occurred in the chloroplast genome were transversion substitutions. In the current study, the Ts/Tv ratio was found to be 0.49, which indicates higher transversion substitutions compared to transition substitutions and is also in agreement with previous studies of angiosperm chloroplast genomes in which the authors reported Ts/Tv < 1 [36,45,54].

Commonly used molecular markers, including ITS1, ITS2, *matK*, *trnH*-*psbA*, *rpoC1*, *rbcL*, and *rpoB*, did not well-resolve the phylogenetic relationships among closely related species of *Artemisia* [29,55,56]. Therefore, these loci could not be employed as barcodes. Moreover, certain other limitations make them unsuitable for barcoding, including their problematic, time consuming, and less robust amplification process and inefficient sequencing [57]. The two approaches that have been suggested for the barcoding of plant species are the use of the whole chloroplast genome as a long barcode [57] or use of molecular markers from mutational hotspots [23,57]. The whole chloroplast genome can be the best choice for the barcoding of plant species as it shows significance polymorphism. However, sequencing of the chloroplast genome is not cost-effective and cannot be preferred. Therefore, we followed the second approach, which was also preferred by other researchers [26,36,37,38,45]. Recently, Meng et al. [58] reported mutational hotspots based on a comparative analyses of the chloroplast genome using mVista, whereas Shahzadi et al. [45] reported twenty mutational hotspots based on comparative analyses of four highly diverse species using multiple MAFFT alignment. Shahzadi et al. [45] used a more technical approach. However, they did not utilize certain genomic resources. In the current study, we utilized nine genomes of *Artemisia* by also including those species that were embedded among the diverse species of *Artemisia* and identified twenty mutational hotspots. Among the twenty mutational hotspots, sixteen mutational hotspots were those identified by Shahzadi et al. [45]. However, the mutational hotspots varied in the extent of divergence. The four mutational hotspots that differed from the study of Shahzadi et al. [45] were *psbA*-*trnK*, *trnS*-*rps4*, *psbK*-*psbI*, and *ycf4*-*cemA*. Therefore, our study confirmed the suitability of a few highly diverse species for the identification of suitable polymorphic loci to design cost-effective, authentic, and robust markers.

The inferring of phylogeny revealed that the genus *Artemisia* is a monophyletic genus that is sister to the genus *Chrysanthemum*. A similar result has also been provided by Shahzadi et al. [45] based on the 77 protein-coding sequences of the chloroplast genome.

In summary, our study provides insight into the chloroplast genome structure of the highly medicinal species *A. scoparia*. The genomic features revealed a close resemblance among *Artemisia* species. The identified microsatellite and twenty mutational hotspots might be helpful for studies of population genetics and phylogenetics of the genus *Artemisia*. Moreover, our study confirms the suitability of a few highly diverse species for the identification of suitable polymorphic loci for the development of effective markers.

## 4. Materials and Methods

### 4.1. DNA Extraction and Sequencing

The plant of *Artemisia scoparia* was collected from District Attock in the Potohar region of Pakistan. Leaf tissues that did not show any apparent disease symptoms were collected from the plant of *A. scoparia*. The leaf tissues were washed with 70% ethanol and silica dried for future DNA extraction. Whole genomic DNA was extracted by the DNeasy Plant Mini Kit (Qiagen, Inc., Germany). The quality and quantity of DNA was determined by 1% agarose gel electrophoresis and Multiskan GO (Thermo Scientific Inc., USA). The high-quality DNA was sent to Novogene, Hong Kong and sequenced from paired ends with 150 bp short reads and a 350 bp insert size using HiSeq2500 (Illumina, USA).

### 4.2. Genome Assembly and Annotation

The chloroplast genome of *A. scoparia* was de novo assembled following Abdullah et al. [37], using Kmer values of 121, 111, 91, 71, and 61 in Velvet 1.2.10 [59]. The coverage depth analyses were performed with Burrow wheal aligner (BWA) [60] and boundaries of LSC, SSC, and IR (IRa and IRb) regions were defined by the manual visualization of scaffolds in Geneious R8.1 [61], following Abdullah et al. [37]. The de novo assembled genome was annotated using the GeSeq annotation tool [62], whereas the transfer RNA genes were further verified by tRNAscan-SE version 2.0.3 [63] and ARAGORN v.1.2.38 [64]. The circular diagram of the chloroplast genome was drawn by Organellar Genome DRAW [65]. The required five-column tab-delimited table of genome annotations used to submit our sequence to the National Centre for Biotechnology Information (NCBI) was generated by GB2sequin [66]. GenBank assigned an accession number of MN385624.

### 4.3. Comparative Analyses and Genomic Features

The genomic features of *A. scoparia* were determined in Geneious R8.1 [61], which compared its genomic features with eight other publicly available species of the genus *Artemisia*. The contraction and expansion of inverted repeat regions were also examined among the nine species of the genus *Artemisia* using IRscope [67].

### 4.4. Amino Acid Frequency, Codon Usage, and RNA Editing Sites

The amino acid frequency and codon usage of protein-coding sequences of the chloroplast were determined in *A. scoparia* using Geneious R8.1 [61], whereas putative RNA editing sites in 35 protein-coding genes of cp were determined using PREP [68] (Predictive RNA editor for plants).

### 4.5. Microsatellites and Oligonucleotide Repeats Analyses

We used Perl script MIcroSAtellite identification tools (MISA) [69] to determine microsatellites in the cp genome of *A. scoparia*. The parameters adjusted for microsatellite detection included 10 for mono-; 5 for di-; 4 for tri-; and 3 each for tetra-, penta-, and hexanucleotide repeats.

To detect oligonucleotide repeats, we used the REPuter [70] program to detect four different types of repeats: forward (F), reverse (R), palindromic (P), and complementary (C) repeats. The parameters of the oligonucleotide program included a minimum repeat size of 30 bp with three mismatches to detect repeats with a minimum of 90% similarities.

### 4.6. Substitutions and InDel Analyses

We determined the numbers and types of substitutions along with the InDels event in each region of the chloroplast genome. We used *A. capillaris* as a reference for *A. scoparia* following Shahzadi et al. [45] and pairwise aligned IR, LSC, and SSC regions of both genomes using MAFFT v.5 (Multiple Alignment with Fast Fourier Transform) [71]. The numbers and types of substitutions were described in Geneious R8.1 [61]. The numbers of InDels events were determined by analysing the pairwise alignment of each region in DnaSP v.5.10 [72].

### 4.7. Screening of Divergence Regions

We determined the extent of divergence of the protein-coding sequences, intronic regions, and intergenic spacer regions. For this purpose, we multiple aligned nine species of the genus *Artemisia* by MAFFT [71] and counted the number of substitutions and InDels events within each region by visualizing them in Geneious R8.1 [61], following Shahzadi et al. [45]. The nine species that were included in the comparison for the determination of divergence regions are provided in Figure 2.

### 4.8. Phylogeny in the Genus Artemisia and Family Asteraceae

We inferred the phylogeny of the genus *Artemisia* and family Asteraceae among thirty species following the methodological approach of Shahzadi et al. [45] and reconstructed the maximum likelihood tree using the IQ-TREE [73,74,75] program, whereas TreeDyn was used to enhance the phylogenetic tree visualization and analyses [76,77].

## Figures and Tables

**Table 1:** Genomic features of *Artemisia scoparia*.

Characteristics		*Artemisia scoparia*
**Size (Base Pair; bp)**		151,060
IR length (bp)		24,972
LSC length (bp)		82,834
SSC length (bp)		18,282
Number of genes		114
Duplicate genes		18
tRNA genes		30
rRNA genes		4
Protein-coding genes		80
GC content	Total (%)	37.5
	CDS (%)	37.8
	IR (%)	43.1
	LSC (%)	35.6
	SSC (%)	30.8
	rRNA (%)	55
	tRNA (%)	52.7
	All gene %	39.3

**Table 2:** Microsatellites in *Artemisia scoparia*.

Repeats	3	4	5	6	7	8	9	10	11	12	13	14	15	16	17	18	19	20	Total
A	-	-	-	-	-	-	-	10	2	5									17
T	-	-	-	-	-	-	-	11	3	1	3	2	1			1		1	23
AT	-	-	3	2	1														6
TA	-	-	2		1														3
ATA	-	1																	1
ATT	-	1																	1
TAA	-		1																1
TTA	-	1																	1
TTC	-	1																	1
AATA	2																		2
AATC	2																		2
AATT	1																		1
ATTT	1																		1
CAAT	1																		1
TAAT	1																		1
TATT	1																		1
TTTA	1																		1
TTTC		1																	1
AAATT	1																		1

**Table 3:** Oligonucleotide repeats in *Artemisia scoparia*.

S.No	Type	Size	Regions	IGS/CDS/Intron	1st Position	2nd Position	Sequence
1	F	32	LSC	psaB/psaA	37,977	40,201	AGAAAAATAAATGCAATAGCTAAATGATGATG
2	F	30	LSC	psaB/psaA	37,988	40,212	TGCAATAGCTAAATGATGATGAGCCATATC
3	R	30	LSC	psaA-ycf3/rps4-trnT	41,744	45,790	ATAAAAAAAAAAAAGATATATATCTAATAT
4	F	41	LSC/IR	ycf3 intron/rps12-ycf15	42,974	96,836	TACAGAACCGTACATGAGATTTTCATCTCATACGGCTCCTC
5	F	39	LSC/SSC	ycf3 intron/ndhA intron	42,976	118,122	CAGAACCGTACATGAGATTTTCATCTCATACGGCTCCTC
6	F	35	LSC/IR	ycf3 intron/ndhB intron	42,979	93,787	AACCGTACATGAGATTTTCATCTCATACGGCTCCT
7	F	30	LSC	rbcL*-rbcL-accD	56,187	56,211	AAAAGAGATAAGGTTCGTTCTCTTAAAAGA
8	F	30	LSC/IR	psaJ-rpl33/ycf15-trnV	66,253	97,999	TAAGAGGATAGCAAGTTACAAATTCTATTT
9	P	48	LSC	psbT-psbN	72,973	72,973	AATTGAAGTAATGAGCCTCCCAATATTGGGAGGCTCATTACTTCAATT
10	P	31	IR	ycf2	86,489	86,489	CGAGAAGCAGATGATTAATCATCTGCTTCCG
11	F	60	IR	ycf2	90,062	90,080	CGATATTGATGAGATTGACGATATTGATGCTAGTGACGATATTGATGCTAGTGACGATAT
12	F	39	IR/SSC	rps12-trnV/ndhA intron	96,838	118,122	CAGAACCGTACATGAGATTTTCACCTCATACGGCTCCTC
13	F	34	IR	rrn4.5-rrn5	105,729	105,761	CATTGTTCAACTCTTTAACAACATGAAAAAACCATTGTT
14	P	43	SSC	ndhD-psaC	115,012	115,012	AAAACATGTGCCCAAAAATAAGATATTTTTGGGCACATGTTTT

* represents copy of repeat shared between genic and intergenic spacer regions.

**Table 4:** Substitutions and InDels events in the chloroplast genome of *Artemisia scoparia*.

Substitution Type	LSC	SSC	IR
A/C	15	5	1
A/G	12	2	2
G/T	16	11	0
C/T	9	1	3
C/G	5	2	0
A/T	2	2	0
Total	59	23	6
No’s of InDels	42	9	3
Average size	3.86	3.11	3.67

**Table 5:** Mutational hotspots in the genus *Artemisia*.

Serial No	Region	Nucleotide Diversity	Total No’s of Mutations	Region Length
1	*ccsA-ndhD*	0.054545	12	220
2	*trnH-psbA*	0.047382	19	401
3	*ndhG-ndhI*	0.042553	16	376
4	*rps18-rpl20*	0.042403	12	283
5	*rps15-ycf1*	0.042056	18	428
6	*trnC-GCA-petN*	0.038917	23	591
7	*rpl32-trnL*	0.037443	39	908
8	*rpoC2-rps2*	0.035433	9	254
9	*trnK-UUU-rps16*	0.035267	31	879
10	*ndhF-rpl32*	0.033915	35	1032
11	*psbA-trnK-UUU*	0.033654	7	208
12	*ndhC-trnV*	0.033646	43	1278
13	*trnS-GGA-rps4*	0.032934	11	334
14	*petN-psbM*	0.032847	18	548
15	*rps16-trnQ-UUG*	0.031729	29	914
16	*trnS-GCU-trnC-GCA*	0.031291	24	767
17	*accD-psaI*	0.03071	16	521
18	*psbK-psbI*	0.030534	12	393
19	*rbcL-accD*	0.030019	16	533
20	*ycf4-cemA*	0.029091	8	275

## Supplementary Materials

The following are available online at https://www.mdpi.com/2223-7747/8/11/476/s1. Table S1: Gene content of the chloroplast genome of *Artemisia scoparia*, Table S2: Analyses of codon usage in *A. scoparia*, Table S3: RNA editing sites in *A. scoparia*, Table S4: Comparison of chloroplast genome features of eight other *Artemisia* species Table S5: Microsatellite loci in *Artemisia scoparia*, Table S6: Species used when inferring phylogeny, Figure S1: Amino acid frequency in *Artemisia scoparia*.
