# Supplementary material for: Chloroplast Genome Sequence of Artemisia scoparia: Comparative Analyses and Screening of Mutational Hotspots
Source: Plants (Basel). 2019 Nov 6;8(11):476. doi: 10.3390/plants8110476 (PMC6918244; doi:10.3390/plants8110476)
Supplement: Supplementary file 1 [file plants-08-00476-s001.zip › plants-610535-supplementary/Supplementary data of artemisia scoparia.docx]

**Table S1:** Gene content of chloroplast genome of *Artemisia scoparia*

| **Classification of genes** | **Subclassification of genes based on functions** | **Gene name** | | | | | **Total numbers** |
| --- | --- | --- | --- | --- | --- | --- | --- |
| Photosynthesis related genes | PhotosystemⅠ | *psaA* | *psaB* | *psaC* | *psaI* | *psaJ* | 5 |
|  | PhotosystemⅡ | *psbA* | *psbB* | *psbC* | *psbD* | *psbE* | 15 |
|  |  | *psbF* | *psbH* | *psbI* | *psbJ* | *psbk* |  |
|  |  | *psbL* | *psbM* | *psbN* | *psbT* | *psbZ* |  |
|  | Cytochrome  b/f compelx | *petA* | *petB** | *petD** | *petG* | *petL* | 6 |
|  |  | *petN* |  |  |  |  |  |
|  | ATP synthase | *atpA* | *atpB* | *atpE* | *atpF** | *atpH* | 6 |
|  |  | *atpI* |  |  |  |  |  |
|  | NADPH dehydrogenase | *ndhA** | *ndhB^a^,** | *ndhC* | *ndhD* | *ndhE* | 12 |
|  |  | *ndhF* | *ndhG* | *ndhH* | *ndhI* | *ndhJ* |  |
|  |  | *ndhK* |  |  |  |  |  |
|  | Rubisco | *rbcl* |  |  |  |  | 1 |
| Self-replication | Transcription | *rpoA* | *rpoB* | *rpoC1** | *rpoC2* |  | 4 |
|  | Small subunit  of ribosome | *rps2* | *rps3* | *rps4* | *rps7^a^* | *rps8* | 13 |
|  |  | *rps11* | *rps12^b^,^**^* | *rps14* | *rps15* | *rps16* |  |
|  |  | *rps18* | *rps19* |  |  |  |  |
|  | Large subunit  of ribosome | *rpl2^a^,** | *rpl14* | *rpl16** | *rpl20* | *rpl22* | 11 |
|  |  | *rpl23^a^* | *rpl32* | *rpl33* | *rpl36* |  |  |
|  | Translational initiation factor | *infA* |  |  |  |  | 1 |
|  | Ribosomal RNA | *rrn4.5^a^* | *rrn5 ^a^* | *rrn16^a^* | *rrn23^a^* |  | 8 |
|  | Transfer RNA | *trnI-CAU^a^* | *trnL-CAA^a^* | *trnV-GAC^a^* | *trnI-GAU^a,^** | *trnA-UGC^a,^** | 37 |
|  |  | *trnR-ACG^a^* | *trnN-GUU^a^* | *trnL-UAG* | *trnP-UGG* | *trnW-CCA* |  |
|  |  | *trnM-CAU* | *trnV-UAC^*^* | *trnF-GAA* | *trnL-UAA^*^* | *trnT-UGU* |  |
|  |  | *trnS-GGA* | *trnfM-CAU* | *trnG-UCC^a,*^* | *trnS-UGA* | *trnT-GGU* |  |
|  |  | *trnR-UCU* | *trnE-UUC* | *trnY-GUA* | *trnD-GUC* | *trnC-GCA* |  |
|  |  | *trnS-GCU* | *trnQ-UUG* | *trnK-UUU** | *trnH-GUG* |  |  |
| 0ther genes | Cytochrome c synthesis | *ccsA* |  |  |  |  | 1 |
|  | RNA processing | *matK* |  |  |  |  | 1 |
|  | Carbon metabolism | *cemA* |  |  |  |  | 1 |
|  | Fatty acid synthesis | *accD* |  |  |  |  | 1 |
|  | proteolysis | *clpP*** |  |  |  |  | 1 |
| Genes of unknown function | | *Ycf1* | *ycf2 ^a^* | *ycf3**,* | *ycf4* | *ycf15^a^* | 7 |
| Pseudogene | | *Ycf1^ψ^* |  |  |  |  | 1 |
| Total genes | | | | | | | 133 |

***^a^*** represents the duplicated genes in IR regions, * represents the genes with one intron, and ** represents the gene with two introns and, **^b^** represents the gene divided into two independent transcription units.

Table S2. Analyses of codon usage in *A. scoparia*

| Codons | Amino acid | No’s of codons | Codons | Amino acid | No’s of codons |
| --- | --- | --- | --- | --- | --- |
| GCA | A | 828 | CCA | P | 658 |
| GCC | A | 459 | CCC | P | 378 |
| GCG | A | 307 | CCG | P | 318 |
| GCT | A | 1239 | CCT | P | 885 |
| TGC | C | 172 | CAA | Q | 1478 |
| TGT | C | 409 | CAG | Q | 470 |
| GAC | D | 425 | AGA | R | 973 |
| GAT | D | 1729 | AGG | R | 342 |
| GAA | E | 1002 | CGA | R | 688 |
| GAG | E | 689 | CGC | R | 211 |
| TTC | F | 1012 | CGG | R | 243 |
| TTT | F | 1985 | CGT | R | 702 |
| GGA | G | 1398 | AGC | S | 250 |
| GGC | G | 378 | AGT | S | 827 |
| GGG | G | 612 | TCA | S | 841 |
| GGT | G | 1170 | TCC | S | 654 |
| CAC | H | 293 | TCG | S | 330 |
| CAT | H | 958 | TCT | S | 1198 |
| ATA | I | 1404 | ACA | T | 823 |
| ATC | I | 873 | ACC | T | 492 |
| ATT | I | 1176 | ACG | T | 253 |
| AAA | K | 1095 | ACT | T | 1059 |
| AAG | K | 738 | GTA | V | 1084 |
| CTA | L | 728 | GTC | V | 349 |
| CTC | L | 394 | GTG | V | 376 |
| CTG | L | 385 | GTT | V | 1025 |
| CTT | L | 1256 | TGG | W | 922 |
| TTA | L | 1791 | TAC | Y | 366 |
| TTG | L | 1167 | TAT | Y | 1625 |
| ATG | M | 1260 | TAA | * | 98 |
| AAC | N | 575 | TAG | * | 42 |
| AAT | N | 1048 | TGA | * | 33 |


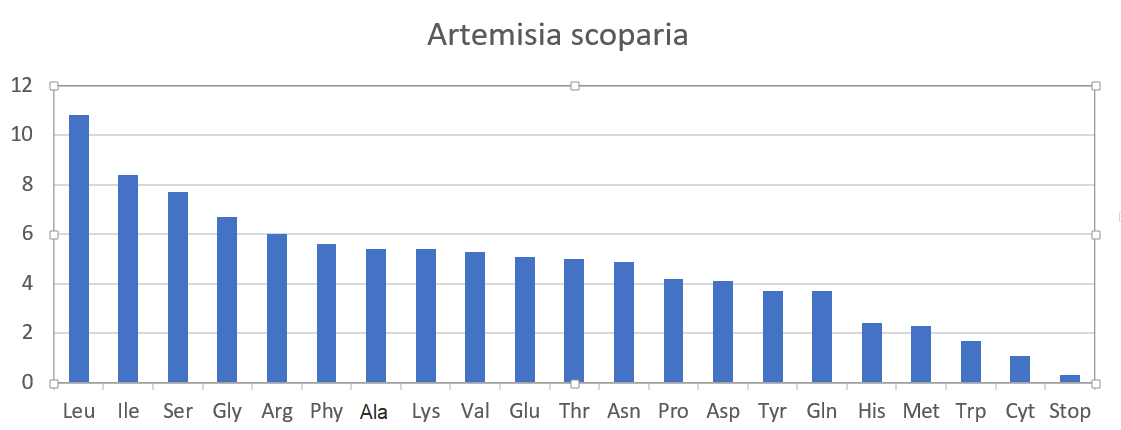


Figure S1. Amino acids frequency in *Artemisia* *scoparia*

Table S3. RNA editing sites in *A. scopraria*

| **Gene** | **Nucleotide positon** | **Amino acid position** | **Codon and amino acid conversion** | **Score** |
| --- | --- | --- | --- | --- |
| *accD* | 125 | 42 | ACG (T) => ATG (M) | 1 |
|  | 370 | 124 | CCT (P) => TCT (S) | 1 |
|  | 1231 | 411 | CCA (P) => TCA (S) | 1 |
|  | 1439 | 480 | CCT (P) => CTT (L) | 1 |
| *atpA* | 773 | 258 | TCA (S) => TTA (L) | 1 |
| *atpB* | 1469 | 490 | TCG (S) => TTG (L) | 1 |
| *atpI* | 629 | 210 | TCG (S) => TTG (L) | 1 |
| *ccsA* | 110 | 37 | CCA (P) => CTA (L) | 0.86 |
|  | 376 | 126 | CCC (P) => TCC (S) | 0.86 |
| *matK* | 284 | 95 | TCT (S) => TTT (F) | 0.86 |
|  | 325 | 109 | CCT (P) => TCT (S) | 1 |
|  | 643 | 215 | CAT (H) => TAT (Y) | 1 |
|  | 1246 | 416 | CAT (H) => TAT (Y) | 1 |
| *ndhA* | 107 | 36 | CCT (P) => CTT (L) | 1 |
|  | 566 | 189 | TCA (S) => TTA (L) | 1 |
|  | 1073 | 358 | TCC (S) => TTC (F) | 1 |
| *ndhB* | 149 | 50 | TCA (S) => TTA (L) | 1 |
|  | 467 | 156 | CCA (P) => CTA (L) | 1 |
|  | 586 | 196 | CAT (H) => TAT (Y) | 1 |
|  | 611 | 204 | TCA (S) => TTA (L) | 0.8 |
|  | 737 | 246 | CCA (P) => CTA (L) | 1 |
|  | 746 | 249 | TCT (S) => TTT (F) | 1 |
|  | 830 | 277 | TCA (S) => TTA (L) | 1 |
|  | 836 | 279 | TCA (S) => TTA (L) | 1 |
|  | 1481 | 494 | CCA (P) => CTA (L) | 1 |
| *ndhD* | 2 | 1 | ACG (T) => ATG (M) | 1 |
|  | 383 | 128 | TCA (S) => TTA (L) | 1 |
|  | 599 | 200 | TCA (S) => TTA (L) | 1 |
|  | 878 | 293 | TCA (S) => TTA (L) | 1 |
|  | 887 | 296 | CCC (P) => CTC (L) | 1 |
|  | 1310 | 437 | TCA (S) => TTA (L) | 0.8 |
| *ndhF* | 290 | 97 | TCA (S) => TTA (L) | 1 |
| *ndhG* | 166 | 56 | CAT (H) => TAT (Y) | 0.8 |
|  | 314 | 105 | ACA (T) => ATA (I) | 0.8 |
| *petB* | 430 | 144 | CGG (R) => TGG (W) | 1 |
|  | 623 | 208 | CCA (P) => CTA (L) | 1 |
| *psbF* | 77 | 26 | TCT (S) => TTT (F) | 1 |
| *psbL* | 2 | 1 | ACG (T) => ATG (M) | 1 |
| *rpl20* | 308 | 103 | TCA (S) => TTA (L) | 0.86 |
| *rpoA* | 824 | 275 | TCA (S) => TTA (L) | 1 |
| *rpoB* | 983 | 328 | GCT (A) => GTT (V) | 1 |
| *rpoC1* | 508 | 170 | CCC (P) => TCC (S) | 1 |
|  | 799 | 267 | CGT (R) => TGT (C) | 1 |
|  | 1589 | 530 | GCA (A) => GTA (V) | 0.86 |
|  | 2042 | 681 | CCA (P) => CTA (L) | 1 |
| *rpoC2* | 1972 | 658 | CTT (L) => TTT (F) | 0.83 |
|  | 2737 | 913 | CCC (P) => TCC (S) | 1 |
|  | 3728 | 1243 | TCG (S) => TTG (L) | 0.86 |
| *rps2* | 248 | 83 | TCA (S) => TTA (L) | 1 |
| *rps14* | 80 | 27 | TCA (S) => TTA (L) | 1 |
|  | 149 | 50 | CCA (P) => CTA (L) | 1 |

Table S4. Comparison of chloroplast genome features of other eight *Artemisia* species

| **Species** | **Genome size**  **(bp)** | **LSC**  **(bp)** | **IR**  **(bp)** | **SSC**  **(bp)** | **Total number of genes** | **CDS** | **rRNA** | **tRNA** | **GC**  **%** | **Accession** |
| --- | --- | --- | --- | --- | --- | --- | --- | --- | --- | --- |
| ***A. absinthium*** | 151,193 | 82,982 | 24,894 | 18,423 | 114 | 80 | 4 | 30 | 37.4 | MK188885 |
| ***A. frigida*** | 151,076 | 82,740 | 24,972 | 18,392 | 114 | 80 | 4 | 30 | 37.5 | JX293720 |
| ***A. fukudo*** | 151,011 | 82,751 | 24,956 | 18,348 | 114 | 80 | 4 | 30 | 37.5 | MK569048 |
| ***A. montana*** | 151,130 | 82,873 | 24,959 | 18,339 | 114 | 80 | 4 | 30 | 37.5 | KF887960 |
| ***A. gmelinii*** | 151,318 | 83,061 | 24,961 | 18,335 | 114 | 80 | 4 | 30 | 37.5 | KY073390 |
| ***A. capillaris*** | 151,056 | 82,821 | 24,963 | 18,309 | 114 | 80 | 4 | 30 | 37.5 | KU736963 |
| ***A. argyi*** | 151,192 | 82,930 | 24,959 | 18,344 | 114 | 80 | 4 | 30 | 37.5 | KM386991 |
| ***A. maritima*** | 151,061 | 82,801 | 24,958 | 18,344 | 114 | 80 | 4 | 30 | 37.4 | MK532038 |

Table S6. Species used in inferring of phylogeny

| **Species** | **Gene bank accession number** |
| --- | --- |
| *Artemisia argyi* | NC_030785 |
| *Artemisia montana* | NC_025910 |
| *Artemisia gmelinii* | NC_031399 |
| *Artemisia frigida* | NC_020607 |
| *Artemisia fukudo* | KU360270 |
| *Artemisia annua* | MF623173 |
| *Artemisia capillaris* | NC_031400 |
| *Artemisia selengensis* | MH042532 |
| *Artemisia absinthium* | MK188885 |
| *Artemisia maritima* | MK532038 |
| *Ambrosia trifida* | NC_036810 |
| *Ambrosia artemisiifolia* | MG019037 |
| *Chrysanthemum boreale* | NC_037388 |
| *Chrysanthemum X morifolium* | NC_020092 |
| *Chrysanthemum indicum* | NC_020320 |
| *Cynara cardunculus var. scolymus* | KM035764 |
| *Cynara cornigera* | KP842707 |
| *Cynara baetica* | KP842706 |
| *Dendrosenecio mereuensis* | MG560049 |
| *Dendrosenecio kilimanjari* | MG560045 |
| *Dendrosenecio cheranganiensis* | MG560044 |
| *Diplostephium oblanceolatum* | NC_034830 |
| *Diplostephium jenesanum* | NC_034829 |
| *Diplostephium hippophae* | NC_034831 |
| *Helianthus tuberosus* | MG696658 |
| *Helianthus debilis* | NC_030173 |
| *Helianthus argophyllus* | NC_030275 |
| *Saussurea polylepis* | NC_036490 |
| *Saussurea chabyoungsanica* | NC_036677 |
